# Which serum markers predict the success of reimplantation after periprosthetic joint infection?

**DOI:** 10.1186/s10195-022-00664-5

**Published:** 2022-09-16

**Authors:** Hongyi Shao, Tao Bian, Yixin Zhou, Yong Huang, Yang Song, Dejin Yang

**Affiliations:** Department of Orthopedic Surgery, Beijing Jishuitan Hospital, Fourth Clinical College of Peking University, Beijing, 100035 China

**Keywords:** Periprosthetic joint infection, Total joint arthroplasty, C-reactive protein, Erythrocyte sedimentation rate, D-dimer

## Abstract

**Purpose:**

In clinical practice, serum C-reactive protein (CRP) and erythrocyte sedimentation rate (ESR) levels are routinely used to screen for periprosthetic joint infection (PJI), but the effectiveness of predicting the success of reimplantation is variable. This study aimed to evaluate the diagnostic effectiveness of serum CRP, ESR, plasma D-dimer, and fibrinogen values in groups achieving treatment success or failure for PJI.

**Methods:**

A total of 119 PJI cases between January 2012 and January 2017 were identified and included in this study. The most recent serum CRP, ESR, plasma D-dimer, and fibrinogen values obtained prior to performing second-stage revision or spacer exchange were collected for analysis. Treatment failure was defined as having been unable to undergo reimplantation due to clinically persistent infection or reinfection after reimplantation.

**Results:**

All these tests showed significantly lower values in the treatment success group than in the treatment failure group. The optimal cutoff serum CRP, ESR, plasma D-dimer, and fibrinogen levels for predicting the success of reimplantation were 9.4 mg/L, 29 mm/h, 1740 ng/mL, and 365.6 mg/dL, respectively. All tests had the same sensitivity (72.7%) except for ESR (63.6%), while their specificities were 92.6%, 88.0%, 72.3%, and 83.2%, respectively. Plasma fibrinogen had the highest AUC value of 0.831 [95% confidence interval (CI), 0.685 to 0.978], followed by serum CRP (0.829) and ESR (0.795); plasma D-dimer had the lowest AUC value of 0.716 (95% CI, 0.573 to 0.859).

**Conclusion:**

Plasma CRP and fibrinogen are good tests for predicting reimplantation success after two-stage revision procedures for patients with PJI.

## Introduction

Periprosthetic joint infection (PJI) is a debilitating complication after total joint arthroplasty (TJA) [1–4]. A two-stage revision procedure remains the most commonly utilized treatment method for PJI [5]. The treatment protocol includes thorough debridement, insertion of an antibiotic-impregnated spacer, and then antibiotic use followed by reimplantation.

Serum C-reactive protein (CRP) and erythrocyte sedimentation rate (ESR) levels are simple and inexpensive tests that are commonly used to screen for PJI [6–8]. Several previous studies have addressed the importance of these tests in the setting of two-stage revision procedures for PJI [9, 10]. However, the results have been variable due to multiple factors, including the interval time between two stages, different reference standards, and a limited number of cases. Synovial white blood cell (WBC) count [9], aspiration culture [11], histological analysis [12], or spacer sonication fluid culture [13] have also been studied with respect to predicting the success of reimplantation. Despite this, there is currently no optimal test available to predict treatment success with reimplantation procedures. Moreover, some tests are difficult to obtain owing to the possibility of a “dry tap” prior to surgery, and other tests require extended waiting periods due to incubation or culture. A definitive serological test would be reliable, quick, and precise. Shahi et al. [14] reported that serum D-dimer was a promising marker for diagnosing PJI and may also be useful in determining the optimal timing for reimplantation. However, a recent study showed D-dimer was not so reliable, while plasma fibrinogen was found to be better for diagnosing PJI. Until now, there has been no study focusing on these serum or plasma biomarkers for evaluation before reimplantation during two-stage revision.

The aim of this study was to investigate whether serum CRP, ESR, D-dimer, and plasma fibrinogen values were different between groups achieving treatment success or failure and to determine the optimal cutoff values of serum CRP, ESR, D-dimer, and plasma fibrinogen for predicting clinical success after reimplantation. We also evaluated the diagnostic effectiveness of each test for predicting the success of reimplantation.

## Patients and methods

### Study design and eligibility criteria

After institutional ethics committee approval, we performed a retrospective study using a registry database of revision total hip and knee arthroplasty procedures performed for PJI at one hospital. Patients treated between January 2012 and January 2017 were screened consecutively. Patients with an antibiotic-impregnated cement spacer who underwent a second-stage operation of a two-stage revision procedure for PJI were included in the study. The exclusion criteria were the following: (1) the index total joint arthroplasty was performed in patients with systemic inflammatory diseases, such as rheumatoid arthritis, ankylosing spondylitis, and systemic lupus erythematosus; (2) patients had venous thromboembolism (VTE), including pulmonary embolism (PE) or deep venous thrombosis (DVT), 6 weeks before reimplantation or reassessment; and (3) other hematological or cardiovascular diseases requiring anti-thrombotic medication. After applying the inclusion and exclusion criteria, 119 patients comprised the patient cohort for the current study. All patients met the MSIS diagnostic criteria [15] for PJI before performing the first-stage revision. There were 54 male patients (45.4%), 65 female patients, and 59 (49.6%) hips and 60 knees (Table 1). The median age of the overall population was 63.0 [interquartile range (IQR), 55.0 to 69.0] years and the median body mass index (BMI) was 25.6 (IQR, 23.4 to 27.8) kg/m^2^ at the time of the first-stage revision. The median time interval between the first- and the second-stage revision procedures was 147 (IQR, 104 to 190) days.

**Table 1:** Demographic data

	Successful (*n *= 108)	Failed (*n* = 11)	*p* value
Sex (no. of patients)			0.543
Male	48	6	
Female	60	5	
Age (years)	65 (55.0 to 69.0)	59 (44.0 to 63.0)	0.023^a^
BMI (kg/m^2^)	25.6 (23.4 to 28.2)	24.8 (23.3 to 27.5)	0.457
Joint type (no. of patients)			0.774
Knee	54	6	
Hip	54	5	
Time interval between the two stages of revision (days)	140.0 (98.0 to 185.5)	154.0 (126.0 to 245.0)	0.313

Median values are shown with interquartile ranges in parentheses

*BMI* body mass index

^a^means the data has statistical difference

### Perioperative management

All the patients underwent a two-stage revision protocol. All prosthetic components, cement, and sequestrum were removed during the first-stage revision procedure. After careful debridement and thorough lavage, an antibiotic-impregnated spacer was inserted. Antibiotic selection was based on preoperative culture results or empirical use for preoperative culture-negative cases. Antibiotic therapy consisted of 2 weeks of intravenous antibiotics, followed by 4 weeks of oral antibiotics. Serum CRP, ESR, and renal function tests were performed every week during this 6-week period of time. Prior to the second-stage revision procedure (i.e., reimplantation), we obtained CRP and ESR values again to assess infection control. Since plasma D-dimer and fibrinogen values were also utilized to assess the patients’ hemodynamic status before surgery, we obtained their values before the second surgery. The plasma D-dimer and fibrinogen were measured using the INNOVANCE^®^ immunoturbidimetry kit (Siemens Healthcare Diagnostics Products GmbH, Marburg, Germany). The postoperative symptoms, signs, and laboratory tests of patients were evaluated to decide whether the infection was controlled. A final reconstruction in the form of revision prostheses was performed for 113 patients with controlled infection, while six patients were diagnosed with persistent infection and underwent repeat spacer exchange. The latest serum CRP, ESR, D-dimer, and plasma fibrinogen values collected prior to the second-stage revision or spacer exchange were analyzed. All the patients were routinely followed up postoperatively. If the event a patient did not present to the outpatient clinic, we contacted them regarding their status of infection.

### Outcome measurements

Treatment failure was defined as having been unable to undergo reimplantation due to a clinically persistent infection that met the MSIS criteria or a case that failed after reimplantation. Diaz-Ledezma et al. [16] described reimplantation failure as evidence of (1) a fistula, drainage, or pain, and infection recurrence caused by the same organism; (2) subsequent surgical intervention for infection after reimplantation surgery; or (3) the occurrence of PJI-related mortality. This definition enabled us to include not only cases of apparent persistent infection that were diagnosed before reimplantation, but also cases with undetected persistent infection and introduced reinfection after reimplantation.

### Statistical analysis

We used Fisher’s exact test to analyze categorical variables and the Mann–Whitney* U* test to analyze continuous variables. The ability of the serum CRP, ESR, D-dimer, and fibrinogen values to predict subsequent failure was evaluated with receiver operating characteristic (ROC) curves. The optimum cutoffs for the serum CRP, ESR, D-dimer, and fibrinogen values to predict subsequent failure were determined when Youden’s* J* statistic was maximal (Youden’s* J* = sensitivity + specificity − 1). The corresponding sensitivities, specificities, accuracy, and positive and negative predictive values were calculated for the serum CRP, ESR, D-dimer, and fibrinogen values using the optimum cutoff values. We calculated AUC values and 95% confidence intervals (CIs). The discriminatory values of curves were interpreted as excellent (0.9 to 1), good (0.8 to 0.89), fair (0.7 to 0.79), poor (0.6 to 0.69), or no discriminatory capacity (0.5 to 0.59). Statistical analyses were performed using SPSS version 24.0 (SPSS Inc., Chicago, IL, USA) and MedCalc software version 15.2.2 (Mariakerke, Belgium). A *p*-value of less than 0.05 was considered statistically significant.

## Results

Of the 119 patients, 108 (90.8%) achieved clinical success at a minimum 2-year follow-up after reimplantation, while 11 (9.2%) had treatment failure. Except for the patients of a younger age in whom treatment failed, there were no differences in the demographic data between the treatment success or failure groups (Table 1). In a comparison of the median ESR, CRP, D-dimer, and fibrinogen values between the treatment success and failure groups, all the values were found to be significantly lower in the treatment success group by comparison to the treatment failure group (Table 2).

**Table 2:** Comparison of values between patients in the treatment success group and patients in the treatment failure group

	Successful (*N* = 108)	Failed (*N* = 11)	*p* value	Reference range
CRP (mg/L)	4.1 (2.7 to 6.9)	17.6 (4.5 to 64.1)	< 0.001^a^	< 10
ESR (mm/h)	14 (8 to 21)	30 (18 to 58)	0.001^a^	30
D-dimer (ng/mL)	1280 (895 to 2078)^b^	2060 (1160 to 3445)	0.019^a^	< 500
Fibrinogen (mg/dL)	297.3 (252.8 to 345.4)^b^	426.4 (328.0 to 571.5)	< 0.001^a^	200–400

^a^means the data has statistical difference; ^b^in the successful group there were 7 patients had no test result. The values are given as the median with the interquartile range in parentheses

The optimal cutoff values for the CRP, ESR, D-dimer, and fibrinogen tests were 9.4 mg/L, 29 mm/h, 1740 ng/mL, and 365.6 mg/dL, respectively (Table 3). In the time prior to second-stage reimplantation surgery, CRP was the most reliable for ruling in persistent infection, with a specificity of 92.3% (95% CI, 85.5%–96.5%) and a negative predictive value of 97.1% (95% CI, 91.1%–99.2%). The specificity for the serum D-dimer test was 72.3% (95% CI, 62.3%–80.5%), while the specificities of ESR and fibrinogen were higher: 88.0% (95% CI, 80.0%–93.1%) and 83.2% (95% CI, 74.1%–89.6%), respectively. The CRP, D-dimer and fibrinogen tests had the same sensitivity of 72.7% (95% CI, 39.3%–92.7%), while ESR had a lower value of 63.6% (95% CI, 31.6%–87.6%).

**Table 3:** Summary of results

Variable	CRP	ESR	D-Dimer	Fibrinogen
Area under the curve (95%CI)	0.829 (0.668–0.990)	0.795 (0.647–0.943)	0.716 (0.573–0.859)	0.831 (0.685–0.978)
Optimum cutoff value	9.4 mg/L	29 mm/h	1740 ng/mL	365.6 mg/dL
No. of patients				
True negative	100	95	73	84
False negative	3	4	3	3
False positive	8	13	28	17
True positive	8	7	8	8
Sensitivity (%)	72.7 (39.3–92.7)	63.6 (31.6–87.6)	72.7 (39.3–92.7)	72.7 (39.3–92.7)
Specificity (%)	92.6 (85.5–96.5)	88.0 (80.0–93.2)	72.3 (62.3–80.5)	83.2 (74.1–89.6)
Positive predictive value (%)	50.0 (25.5–74.5)	35.0 (16.3–59.1)	22.2 (10.7–39.6)	32.0 (15.7–53.6)
Negative predictive value (%)	97.1 (91.1–99.2)	96.0 (89.4–98.7)	96.1 (88.1–99.0)	96.6 (89.6–99.1)

95% CIs are provided in parentheses

*CRP* C-reactive protein, *ESR* erythrocyte sedimentation rate

**Fig. 1:**
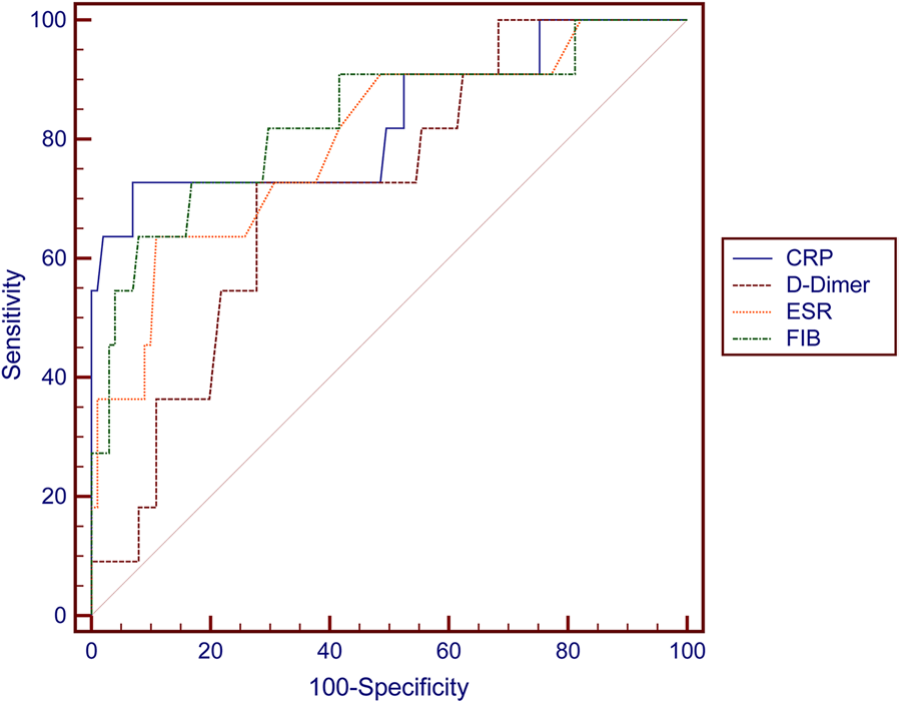
ROC curves of the serum CRP, ESR, plasma D-dimer, and fibrinogen values for predicting the clinical success of the treatment of PJI

The ROCs generated for the four tests had different AUC values (Fig. 1, Table 3). Fibrinogen and CRP had AUCs above 0.8: 0.831 (95% CI, 0.685–0.978) and 0.829 (95% CI, 0.668–0.99), respectively. This indicated they showed good performance when used to evaluate whether or not the infection was controlled before reimplantation. D-dimer had the lowest AUC value of 0.716 (95% CI, 0.573–0.859), while that of ESR was 0.795 (0.647–0.943), which showed that they are of only fair diagnostic value.

## Discussion

### Main findings

To the best of the authors’ knowledge, this represents the first study to focus on preoperative serological tests for predicting the failure of reimplantation using clinical follow-up as a reference standard. In this regard, we found that CRP and plasma fibrinogen values were high in the failed reimplantation group and demonstrated high diagnostic efficiency in predicting persistent infection.

### Comparison with previous studies

After a first-stage debridement is performed, including spacer insertion and following the use of antibiotics, it is difficult to determine whether PJI is controlled [17, 18]. Although previous studies [9, 10, 19] have attempted to utilize CRP and ESR values to evaluate infection status, results have been variable. In fact, the reference standards, including culture, could introduce the possibility of false-positive or -negative results. While the first International Consensus Meeting on PJI previously established an algorithm to diagnose PJI [15], it is not suitable for the diagnosis of persistent infection prior to reimplantation [17]. Clinical results with careful follow-up should represent the gold standard means to retrospectively judge infection control prior to reimplantation. Diaz-Ledezma et al. [16] have previously defined the success criteria for treating PJI. Beyond serum CRP and ESR values, Shahi et al. [14] assessed another possible preoperative serological test to determine the status of infection prior to reimplantation; however, data was insufficient, with limited cases.

In our study, the AUC value of serum CRP is above 0.8, which indicates that this is a reliable test for predicting reimplantation success in the treatment of PJI. Using the optimal cutoff value of 9.4 mg/L, CRP had a specificity of over 90%, while its sensitivity was 72.7%. This result is similar to that obtained when using serum CRP to diagnose PJI [20, 21], suggesting it has limited utility for ruling out persistent infection before second-stage reimplantation surgery. Hoell et al. [22] and Kusuma et al. [10] both reported that the specificity of CRP was above 90%, which was similar to our results. However, compared to our CRP cutoff value, the results from Hoell et al. [22] and Kusuma et al. [10] were much higher: 25 mg/L and 177.5 mg/L, respectively. Due to their use of a high cutoff value, the specificity of serum CRP in those works was increased at the expense of decreased sensitivity, which was reduced to 44% and 13%, respectively. Other studies [19, 23, 24] used 10 mg/L as the cutoff value of serum CRP, which is the value of the MSIS criteria for PJI diagnosis; however, the results obtained were variable. Several studies [10, 19, 23] used ESR to predict persistent infection after the first-stage procedures. The sensitivities were similar to the results of the current study, while the specificities were less, thus revealing that the ability to rule in persistent infection was compromised. Although ESR is a systemic marker of infection, it can be variable due to many factors, including antibiotic use [25], iatrogenic trauma from the first-stage procedure, and the presence of systemic inflammation before the index surgery [26], among others. One study [24] had only 21 cases, which means that there was selection bias due to limited cases. The other studies [10, 19, 23] had a shorter time interval between the two stages than in our study. The first-stage procedure will influence serum CRP for at least 3 weeks and the ESR level for even longer [27], which may compromise accuracy if the time interval is short. The average time interval in our cohort was more than 160 days, which was enough time for the serum CRP and ESR values to decrease after the first-stage revision.

### Implications for clinical practice

Local infection can initiate an inflammatory reaction characterized by vasodilation and increased endothelial permeability, which activates coagulation factors and prevents the spread of microorganisms into the systemic circulation [28]. The coagulation will activate fibrinolysis, while bacteria that overcome the fibrin confinement will also convert plasminogen to plasmin with kallikrein, both of which will elevate the serum D-dimer level [29, 30]. Shahi et al. [14] first reported the utilization of serum D-dimer for diagnosing PJI and its use for predicting persistent infection before reimplantation. In their cohort, there were only 29 reimplantation cases with D-dimer data, of which five had elevated D-dimer levels. Among those, two patients experienced treatment failure after reimplantation and another three patients were still in follow-up. Due to the limited cases and the short time to follow-up, we could not conclude that serum D-dimer was an excellent test to assess prior reimplantation. The current study found that D-dimer had only fair diagnostic efficiency for predicting persistent infection prior to reimplantation. There are several potential reasons for this. First, aside from infection, trauma, soft-tissue injury, and hematoma could induce a high D-dimer value [31]. Second, after the first stage of debridement and spacer insertion, prophylactic anticoagulation medicine is prescribed for DVT. These factors could influence the coagulation state, which infers that the D-dimer is not as precise for predicting persistent infection prior to reimplantation. The use of plasma fibrinogen for diagnosing PJI was first studied by Li et al. Our study was the first to investigate the efficiency of fibrinogen for predicting reimplantation success. A previous study reported that human neutrophils could induce the formation of fibrinogen. Moreover, fibrinogen may mediate neutrophil–endothelial cell adherence in sepsis. Our study showed that the AUC value of fibrinogen was good, and it was routinely analyzed before surgery, so it was able to provide more information before we decided to do reimplantation.

### Limitations

There are several limitations of this study. First, the study had a relatively small sample size of 119 patients, including only 11 patients with clinical failure, which may introduce statistical bias. With respect to the D-dimer and plasma fibrinogen tests, this study is the first to report their utility for predicting reimplantation success or failure. Second, this is a retrospective study, which introduces the drawbacks inherent to all retrospective studies, including the possibility of missing information and heterogeneity among cases in the cohort. However, all cases in the current study were recent cases from a single center with set inclusion criteria, thereby reducing the possibility of confounding. Third, only serologic tests were evaluated, while synovial WBC count, PMN%, and intraoperative tissue culture may have provided further information. Serologic tests provide more convenience in sampling, less pain to the patient, and are not dependent on obtaining a synovial fluid aspirate or intraoperative tissue culture. Finally, clinical results were used as the reference standard, which may have introduced bias into the results. Reinfection could be a new infection after reimplantation, with bacteria that are different from those in a previous infection. Previous reports used alternative reference standards, such as positive cultures [3] or combined tests [23]. However, currently, there is no gold standard method to detect and/or evaluate persistent infection. The Delphi-based consensus is widely accepted as a treatment target [18], which is most important in clinical practice.

In summary, serum CRP and plasma fibrinogen are good tests for predicting the success of reimplantation after two-stage revision procedures for patients with PJI. Further prospective studies with additional cases are needed to determine the utility of these tests and their optimal cutoff values.

## Data Availability

The datasets used and analyzed during the study will be available from the corresponding authors on reasonable request.
